# Assessment of factual recall and higher-order cognitive domains in an open-book medical school examination

**DOI:** 10.1007/s10459-021-10076-5

**Published:** 2021-10-23

**Authors:** D. J. Davies, P. F. McLean, P. R. Kemp, A. D. Liddle, M. J. Morrell, O. Halse, N. M. Martin, A. H. Sam

**Affiliations:** 1 Imperial College School of Medicine, Imperial College London, London, England; 2 National Heart and Lung Institute, Imperial College London, London, England

**Keywords:** Open-book examinations, Assessment, Medical education

## Abstract

Open-book examinations (OBEs) will likely become increasingly important assessment tools. We investigated how access to open-book resources affected questions testing factual recall, which might be easy to look-up, versus questions testing higher-order cognitive domains. Few studies have investigated OBEs using modern Internet resources or as summative assessments. We compared performance on an examination conducted as a traditional closed-book exam (CBE) in 2019 (N = 320) and a remote OBE with free access to Internet resources in 2020 (N = 337) due to COVID-19. This summative, end-of-year assessment focused on basic science for second-year medical students. We categorized questions by Bloom’s taxonomy (‘Remember’, versus ‘Understand/Apply’). We predicted higher performance on the OBE, driven by higher performance on ‘Remember’ questions. We used an item-centric analysis by using performance per item over all examinees as the outcome variable in logistic regression, with terms ‘Open-Book, ‘Bloom Category’ and their interaction. Performance was higher on OBE questions than CBE questions (OR 2.2, 95% CI: 2.14–2.39), and higher on ‘Remember’ than ‘Understand/Apply’ questions (OR 1.13, 95% CI: 1.09–1.19). The difference in performance between ‘Remember’ and ‘Understand/Apply’ questions was greater in the OBE than the CBE (‘Open-Book’ * ‘Bloom Category’ interaction: OR 1.2, 95% CI: 1.19–1.37). Access to open-book resources had a greater effect on performance on factual recall questions than higher-order questions, though performance was higher in the OBE overall. OBE design must consider how searching for information affects performance, particularly on questions measuring different domains of knowledge.

## Introduction

The COVID-19 pandemic has necessitated rapid and unprecedented change in the delivery of medical school examinations (Craig et al., 2020; Eva & Anderson, 2020; Hannon et al., 2020; Mooney et al., 2020; Sam et al., 2020; Zagury-Orly & Durning, 2020). Social distancing requirements and the inability to enforce strict exam conditions have, in many cases, rendered traditional, closed-book exams (CBEs) impossible. In common with many institutions, at Imperial College School of Medicine (ICSM), we rapidly converted examinations in 2020 designed as CBEs into OBEs, sat remotely via an electronic examination portal (Sam et al., 2020). We did not employ any form of remote proctoring and students were allowed access to resources including the Internet, journals and lecture notes during the assessment. This was done for written exams across different year groups, including early-stage students, where the course focuses more on basic science, and late-stage students where the course focuses on clinical reasoning, decision-making and preparation for clinical practice.

From the literature on OBEs, it was difficult to predict how converting a CBE to an OBE would affect students’ performance. One meta-analysis found that access to open-book resources, and particularly testing aids that students had constructed themselves, improved exam performance (Larwin et al., 2013). However, another meta-analysis concluded that performance tended to be better, and students may have prepared more, with CBEs than OBEs (Durning et al., 2016). However, both meta-analyses found studies where open-book resources had opposing effects on performance. Importantly, there was very little evidence from high-stakes summative assessments conducted with access to modern Internet-based resources (Durning et al., 2016), as took place at ICSM in 2020 (Sam et al., 2020).

We recently reported the results of converting an end-of-year summative assessment (meaning students had to pass to progress/graduate) for final-year medical students from a CBE to an OBE. We found that the median mark was the same as the equivalent exam from the previous year (Sam et al., 2020), suggesting that access to open-book resources did not systematically affect performance.

In this study, we took the opportunity to investigate the effects on performance of converting a CBE to an OBE for early-stage medical students, whose course stage focuses more on understanding of basic science. We compared performance on a summative, end-of-year assessment centered around the life cycle and physiological regulation, conducted as a CBE in 2019 and an OBE in 2020.

We predicted that access to open-book resources would be associated with better performance. If true, we thought that this might be explained because performance on questions testing factual recall would be selectively higher (Krarup et al., 1974), compared to questions that tested higher-order domains of knowledge like understanding concepts and applying concepts to new information (Bloom, 1965). In other words, we thought that the effect of open-book resources on exam performance would be different for questions measuring different cognitive domains.

We thought access to open-book resources would affect performance on this exam more so than in our study of final-year students (Sam et al., 2020) for two reasons. Firstly, early-stage students have less experience preparing for and undertaking CBEs, so the conversion to an OBE may have affected their preparation and approach to answering questions more so than more experienced, final-year, students. Secondly, the early course focuses on basic science and basic science exams tend to heavily feature factual recall (Momsen et al., 2010).

We predicted that factual recall questions would be easier in an OBE because students could more easily find the answers by searching open-book resources, particularly using the Internet. On fact-based questions, it is likely to be relatively clear what information is required to answer the question, which will make searching for that information easier (Bell & Ruthven, 2004). Students may then be able to easily find this information online and choose the correct answer without necessarily recalling the information from their knowledge. We predicted that performance on questions requiring students to show understanding of a concept, or apply knowledge to new information, would be less affected by access to open-book resources. This was because the information required to answer the question should be more complex and may be more difficult to find online (Bell & Ruthven, 2004), which may have been particularly limiting in a time-pressured examination.

## Methods

### Ethical approval

The study was granted ethical approval by the Imperial College London Educational Ethics Review Process (EERP2021-009).

### Examination data

We used data from the single-best-answer (SBA) component of a summative assessment centered around the life cycle and physiological regulation. In 2019, this was conducted as a traditional CBE. In 2020, the assessment for this paper was designed as a CBE but converted to an OBE. The 2019 and 2020 assessments were not the same paper; they had different questions, though 16 were the same. Students were informed of the format change 6 weeks prior to the exam. The 2020 exam was written prior to the COVID-19 pandemic. We compared exam performance over the two years.

The exam was performed by second year medical undergraduates. The module comprised mainly basic science with some clinical applications. This was a ‘high-stakes’, summative examination, requiring examinees to pass to progress to the next course year. Each year, the exam contained 128 single best answer (SBA) questions with 4 distractors. The exam was sat as 2 sections with 64 SBAs in each section.

Each exam also contained 14 multi-part short answer questions. We opted not to analyze these questions. The inclusion of a different type of question would mean that performance was affected by factors related to item type. Most studies comparing OBEs and CBEs have used multiple-choice questions (Durning et al., 2016), so we thought our results would be most generalizable if we focused on the most well-investigated question type. We also did not want to assume that access to open-book materials would affect performance on both question types in the same way.

In 2019, 320 examinees completed the exam under closed-book conditions. In 2020, 337 examinees performed the OBE on an online testing platform (Practique, Fry-IT, United Kingdom). This platform was already in use by the medical school for electronic summative and formative written exams since 2017. Using this platform to deliver OBEs online did not incur additional costs. Examinees could perform the OBE on any device with internet access. The platform did not impose any restrictions on other activities such as web browsing.

There was no remote proctoring in place during the exam. To minimise the risk of collusion, examinees were each presented with the questions in a different random order. Examinees were reminded that academic misconduct would contradict the medical school code of conduct and the professional standards outlined by the UK General Medical Council.

Examinees had 2 h 30 min to complete each of the papers in both years, which equated to around 1 min per SBA question. Instructions to examinees were the same across years. In 2020, examinees were advised that additional resources could be used, although this could add additional time constraints to the completion of all questions. Ebel standard setting was used for both exams and performed by senior faculty at ICSM. The pass mark was the same in both years. Students had no prior experience of OBEs within the medical course.

### Categorization by Bloom’s taxonomy

To determine what domain of cognitive knowledge a question was measuring, or the type of information required to answer it (i.e. factual recall, understanding, application), we categorized exam questions using the cognitive domain of Bloom’s taxonomy (Bloom, 1965). Bloom’s taxonomy is a well-established method of categorizing cognitive knowledge (or cognitive processes, in the revised version (Anderson et al., 2001)) in a hierarchy of complexity.

It has been suggested that it is harder to measure higher-order processes like evaluation and synthesis using multiple-choice questions (Haladyna & Rodriguez, 2013; Hancock, 1994; Martinez, 1999). Given the limitations of the SBA format, we expected most questions would fall within the first three domains: ‘Remember’, ‘Understand’ and ‘Apply’ (in order of increasing complexity). ‘Remember’ questions tested the recall of information without requiring deeper understanding. ‘Understand’ questions tested examinees’ abilities to demonstrate understanding of facts or concepts. ‘Apply’ questions tested examinees’ abilities to use their knowledge to solve new problems or use knowledge in new contexts. We predicted higher performance in the OBE compared to the CBE overall, and that this would be greatest for the ‘Remember’ questions (the lowest level of Bloom’s taxonomy), compared to higher-order categories like ‘Understand’ or ‘Apply’.

Two authors (DJD, PFM) independently categorized each question in the SBA exams according to Bloom’s taxonomy. Both raters had attended a workshop course by ICSM on educational theory that included categorizing questions by Bloom’s taxonomy. Questions that had been categorized differently by each rater were then discussed at a consensus meeting and a final category decided.

Due to considerable overlap between boundaries, low numbers of ‘Apply’ questions and no questions from levels 4–6 of Bloom’s taxonomy, we combined ‘Understand’ and ‘Apply’ questions to produce a binary predictor of ‘Remember’ versus ‘Understand/Apply’. We recognize that some evidence suggests no linear relationship between taxonomic level and item difficulty (Cunnington et al., 1996; Hamamoto Filho et al., 2020) and there is limited evidence to support Bloom’s taxonomy as a single, linear hierarchy in this way (Furst, 1981; Haladyna & Rodriguez, 2013; Marzano & Kendall, 2007). However, our main hypothesis was that factual recall questions would be made selectively easier in an OBE, so we considered collapsing the categories appropriate to compare, in effect, recall questions versus questions that required more than recall.

### Statistical analysis

We tested whether the 2019 and 2020 examinations differed in terms of their proportion of Bloom ‘Remember’ questions using Chi-Square test of proportions.

We then analyzed performance of the SBA exam using logistic regression, treating performance on each item as the number of ‘successful trials’ (i.e. answers) out of the number of total ‘trials’ (number of students answering the question), which follows a binomial distribution. Our dependent variable was the set of 256 questions (128 questions per exam, sat as 2 sections each), with performance averaged over all examinees. This ‘item-centric’ approach contrasts with a traditional ‘person-centric’ approach, where an examinee’s performance is averaged over all questions.

We planned a logistic regression with the outcome variable of counts of correct answers out of total answers and two binary predictors: O*pen-Book* (‘open’ vs ‘closed’ book) and *Remember (*‘Remember’ vs ‘Understand/Apply’). We used model comparison to determine which effects to include in the model. A more complex model can always fit the data better, but inclusion of more complex terms may not significantly improve model performance and could make the model unnecessarily complex. We used Bayesian Information Criterion (BIC), a log-likelihood based model fit index that penalizes complexity, so trading off model fit with parsimony. We compared the following models: null model (no effects), *Open-Book* main effect alone, *Bloom Category* main effect alone, and both main effects with interaction. BIC is a relative fit index and lower values indicate better performance. A difference in BIC of > 10 is considered very strong evidence favoring one model over another (Kass & Raftery, 1995). To test significance of effects in the winning model, we calculated odds-ratios and 95% Wald confidence intervals.

## Results

### Examinee-centric and item-centric exam performance

Table 1 shows descriptive statistics for the scores per examinee, averaged across all items. The average student performance was higher for the OBE than the CBE (t = 14.8, df = 570, *p* < 0.0001). Table 1 also shows descriptive statistics for the scores per item, averaged across all examinees. Cronbach’s alpha (Cronbach, 1951) was 0.91 for the CBE and 0.94 for the OBE.

**Table 1:** shows the student-centric and item-centric descriptive statistics for the exam, performed as a closed-book exam (CBE) in 2019 and open-book exam (OBE) in 2020

	Closed-book exam (2019)	Open-book exam (2020)
Candidate-centric descriptive statistics		
N candidates	320	337
Mean score per candidate (standard deviation) (%)	72.1 (11)	85.7 (8.2)
Median score per candidate (median absolute deviation) (%)	74.2 (10.4)	87.5 (4.6)
Range of scores per candidate (%)	22.7–93	35.9–97.7
Interquartile range of scores per candidate (%)	14.9	6.2
Number of Failing Students	19	9
Item-centric descriptive statistics		
N items	128	128
Mean score per item (standard deviation) (%)	72.1 (18.6)	87.0 (13.7)
Median score per item (median absolute deviation) (%)	75.5 (17.4)	92.1 (9.5)
Interquartile range of scores per item (%)	24.2	17.3
Range of scores per item (%)	18.8–99.1	31.8–99.7

### Descriptive statistics

#### Categorization by Bloom’s taxonomy

Table 2 shows the categorization of the CBE and OBE by Bloom’s Taxonomy. As predicted, there were no questions categorized as ‘Analysis’, ‘Synthesis’ or ‘Evaluation’. Inter-rater reliability of first categorizations of both raters, calculated using Cohen’s kappa with squared weights, was 0.782, indicating moderate agreement. Chi-squared test found no evidence of difference in the numbers of ‘Remember’ versus ‘Understand/Apply’ questions (Chi-squared = 0.070, df = 1, p = 0.76) between in the categorizations of the 2019 and 2020 exams.

**Table 2:** shows the categorization of the closed-book exam (CBE) and open-book exam (OBE) by Bloom’s Taxonomy and item-centric average performance per category. Chi-squared test showed no evidence of difference in the numbers of ‘Remember’ versus ‘Understand/Apply’ questions (Chi-squared = 0.070, df = 1, *p* = 0.76) between the 2019 and 2020 exams

Bloom category	N Items CBE (2019)	Mean (SD) performance per category–CBE (2019)	Median (MAD) performance per category–CBE (2019)	N Items OBE (2020)	Mean (SD) performance per category–OBE (2020)	Median (MAD) performance per category–OBE (2020)
Remember	82	73.0 (19.6)	76.7 (17.8)	79	88.7 (14.9)	94.7 (6.2)
Understand	42	70.1 (17.1)	74.4 (17.1)	43	84.8 (11.6)	85.5 (14.5)
Apply	4	73.8 (15.6)	72.8 (16.4)	6	81.3 (8.3)	81.8 (6.6)
Understand/Apply Combined	46	70.4 (16.8)	74.4 (17.1)	49	84.4 (11.2)	84.3 (14.5)

#### Distributions of scores per item by OBE/CBE and Bloom’s levels

**Fig. 1:**
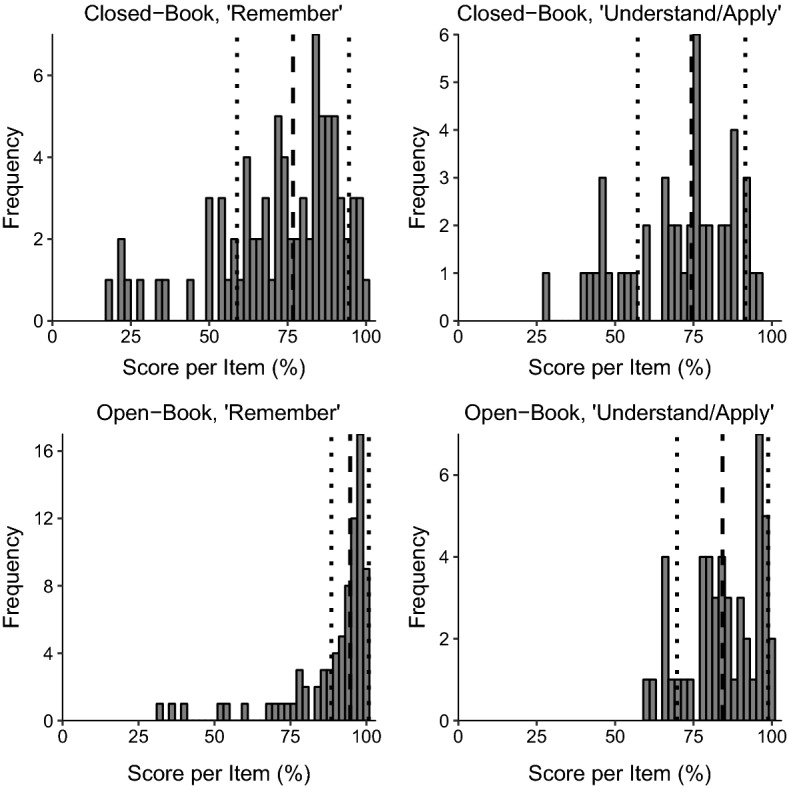
Shows the distributions of percentage scores per item, averaged over all examinees. Items are grouped by their classification by Bloom’s taxonomy (‘Remember’ or ‘Understand/Apply’), and whether they were in the 2019 closed-book exam (CBE) or the 2020 open-book exam (OBE). The thick dashed line shows the median. The thin dashed lines show the interquartile range

Figure 1 shows the distributions of the scores for each item averaged across all examinees, grouped according to the exam format and Bloom level. Descriptively, the mean and median item scores were higher for OBE and for ‘Remember’ questions. We investigated the effects of format and Bloom level further in logistic regressions.

#### Logistic regression model comparison

We fit the following logistic regression models: null model (no effects), *Open-Book* main effect alone, *Bloom Category* main effect alone, and both main effects with interaction.

The best-performing model contained *Open-Book* and *Bloom Category* main effects and their interaction. BIC values are shown in Table 3. The BIC difference indicates ‘Very Strong’ evidence for this model over all others (Kass & Raftery, 1995).

**Table 3:** shows the results of comparing logistic regression models using BIC

Model	BIC
Null (no effects)	17,857.87
Open-book main effect only	14,915.46
Bloom category main effect only	17,747.99
Main effects & interaction	14,731.0

We compared the null model (no effects), to models with a main effect of ‘Open-Book’ (whether the exam was open or closed-book) only, a main effect of ‘Bloom Category’ (whether the item was categorised as ‘Remember’ or ‘Understand/Apply’) only, and a model with ‘Open-Book’, ‘Bloom Category’ main effects and their interaction. The model with the interaction performed the best, indicated by the lowest BIC

#### Effects of open-book versus closed-book on different levels of knowledge

Table 4 shows the results of the winning logistic regression with the interaction between ‘Open-Book’ and ‘Bloom Category’. As predicted, examinees performed better on the OBE, shown by the significant main effect of *Open-Book.* Examinees performed better on ‘Remember’ questions than on ‘Understand/Apply’ questions, shown by the significant main effect of *Bloom Category.* Importantly, there was a greater difference in performance between ‘Remember’ questions and ‘Understand/Apply’ questions in the OBE than the CBE, shown by a significant Open-Book * Bloom Category interaction.

**Table 4:** Shows the results of the winning logistic regression model with predictors of ‘Open-Book’, ‘Bloom Category’ and their interaction

Model	Outcome	Predictor	Log Odds (Standard Error)	Z-value	P-value	Odds Ratio (CI)
Logistic regression model with interaction	N correct answers out of total answers per item	Open-Book	0.82 (0.03)	29.17	< 0.0001	2.26 (95% CI: 2.14—2.39)
		Bloom Category	0.13 (0.02)	5.55	< 0.0001	1.13 (95% CI: 1.09—1.19)
		Open-Book * Bloom Category Interaction	0.25 (0.04)	6.69	< 0.0001	1.28 (95% CI: 1.19—1.37)
Posthoc logistic regression: CBE data only	N correct answers out of total answers per item	Bloom Category	0.13 (0.02)	5.55	< 0.0001	1.13 (99% CI: 1.07—1.2)
Posthoc logistic regression: OBE data only	N correct answers out of total answers per item	Bloom Category	0.37 (0.03)	12.91	< 0.0001	1.45 (99% CI: 1.35—1.56)

Table 4 also shows the results of posthoc logistic regressions performed on open-book and closed-book data separately to understand the interaction term

**Fig. 2:**
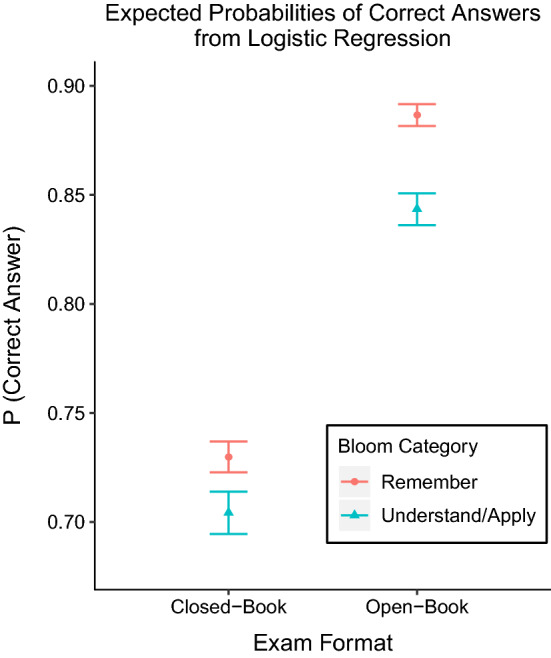
Shows the probabilities of answering a question correctly, extracted from logistic regression models and calculated using the inverse-logit function. Examinees were more likely to correctly answer ‘Remember’ questions than ‘Understand/Apply’ questions, and the gap between ‘Remember’ and ‘Understand/Apply’ questions was greater in the open-book exam. This supported that access to open-book resources made examinees more likely to answer questions correctly, particularly for questions testing recall of facts

To understand the interaction term, we ran two separate post-hoc logistic regressions for the OBE and CBE separately. We used z-tests and Wald confidence intervals as tests of significance, with 99% confidence intervals to adjust for multiple comparisons (Table 4). In each model, a significant effect of Bloom Category showed that performance was higher on ‘Remember’ questions. The OR for correct answers was higher in the OBE than the CBE and the OR had non-overlapping 99% confidence intervals. The OR were converted to probabilities of answering a question correctly using the inverse-logit function and are plotted in Fig. 2. The difference between performance on ‘Remember’ and ‘Understand/Apply’ questions was greater in the OBE than the CBE, as predicted. However, performance was considerably higher for both types of questions in the OBE. We emphasise that the odds of the outcome (number of correct answers out of N examinees answering the question) were generally not rare, and therefore the OR should not be interpreted as an approximation of relative probability.

## Discussion

We compared a summative, end-of-year assessment performed as an OBE in 2020 to the equivalent examination from the previous academic year, performed as a CBE. In this second-year examination focusing on basic science, we found that performance was better overall in the OBE than the CBE and performance on questions testing recall of facts (‘Remember’ in Bloom’s taxonomy) was better than on questions testing higher-order cognitive domains (‘Understand/Apply’ in Bloom’s taxonomy). The difference between performance on ‘Remember’ and ‘Understand/Apply’ questions was greater in the OBE than the CBE, supporting that the access to open-book resources had a greater effect on questions testing factual recall. However, performance on both types of questions improved in the OBE.

We theorized that performance was higher on factual recall questions because answers could more easily be found by accessing information resources, compared to answers to questions at higher levels of Bloom’s taxonomy. Importantly, ours was one of few studies that gave examinees access to modern Internet-based resources in a summative setting (Durning et al., 2016), hopefully making it more authentic to the real-world clinical environment and more transferable to future studies than previous studies of OBEs that limited resource access to textbooks or student-made resources. Access to the Internet is arguably very different from allowing access to a textbook or student-made resources. The Internet can be conceptualized as a ‘transactive memory’ (Goethals, 1987), in which information is outsourced from an individual to other individuals or structures. Through searching the Internet, information can be accessed without needing to know what information is stored or where it is located (Ward, 2013). The Internet is unique as a transactive memory system because it stores virtually all known factual information (Ward, 2013), as opposed to the limitations of a textbook or students’ notes. Further work is necessary to understand how medical students use the Internet in OBEs, and how well we can generalize from older studies to OBEs with online resources.

## The ‘searchability’ of answers and relationship to a taxonomy of cognitive processes

Looking for information to answer a question in an OBE can be conceptualized as an information search task performed by examinees. How ‘searchable’ a question is, i.e. how simple it is to find the answer by searching, is likely to be an important concept in OBE design. If it is very simple to find the correct answer to a question by searching, the less we can be sure that the question is measuring intended learning versus measuring the ability to search. We can try to understand what determines the complexity of searching for information in an SBA by using models from information-searching literature. Numerous models have been advanced to conceptualize information-searching, influenced by computer science, information science and cognitive psychology (Dinet et al., 2012) and there is not consensus on the optimal model.

Campbell (1998) theorized that search complexity is determined by ‘objective’ factors, which are intrinsic to the task and independent of the problem performer, and ‘subjective’ factors of how an individual perceives and interacts with the problem. Objective complexity of a search task depends on its ‘a priori determinability’, which reflects intrinsic uncertainty in the task’s inputs, processes and outputs. We can consider uncertainty in (at least) three domains: (i) what information is required to solve the problem, (ii) how to search for it, and (iii) interpreting the found information (Byström & Järvelin, 1995). There is likely to be an optimal window of objective search complexity in an OBE question, such that it is not made trivial by access to open-book resources, but is not so unclear that the examinee is not sure what is being asked of them.

We argue that the type of knowledge or process required to answer the question (e.g. recall of facts versus understanding of concepts) maps mainly to the first domain of objective complexity: the information required to answer a question. We tried to capture this using Bloom’s taxonomy. The gap in performance between questions testing factual recall versus understanding/application was greater in the OBE than the CBE. Open-book resources may therefore confer a particular advantage to fact-based questions, which may be because there is little uncertainty in the information required to answer them, making the search task less complex (Bell & Ruthven, 2004). For example, a fact-based question, in which examinees must identify which receptor is targeted by a drug, has very clear information requirements and so will be straightforward to look-up.

Uncertainty in the second domain of objective complexity (how to search for required information) in an SBA will be shaped by the information in the question stem and distracters. Uncertainty in how to search has been experimentally manipulated by changing the number and relevance of key terms provided in search problem. Searches have been made more complex by requiring participants to generate their own key words for efficient search queries, rather than providing them (Barsky & Bar-Ilan, 2012; Chevalier et al., 2011; Dommes & Chevalier, 2011; Sanchiz et al., 2017). We suggest that question-writers in OBEs can optimize the complexity of how to search by avoiding key terms, where possible, and requiring examinees to generate their own search terms, such as by requiring them to infer a condition from a clinical presentation rather than give them the condition name, or giving a hemoglobin value rather than stating that a patient is anemic.

The third domain of objective complexity (interpreting the found information) is effectively out of examiner’s control if students are given free Internet access. However, it may be useful during question development to construct searches to answer the question as if one were the examinee, to review what kind of information examinees are likely to find.

Subjective factors affecting search complexity will introduce individual differences in when examinees search, how they search, and how they evaluate and use the found information (Ford et al., 2005). To ensure the fairness of OBEs, it will be important to improve our understanding of individual differences in information-searching. Variability in Internet expertise, even among digital natives, tends to reflect existing social inequalities (Robinson et al., 2015), with poorer expertise associated with factors like lower parental education and socioeconomic deprivation (Hargittai, 2010; Hargittai & Hinnant, 2008). Without careful consideration, we risk introducing ‘digital inequality’ (Hargittai & Hinnant, 2008) into our assessments based on examinees’ searching competencies.

## Why was performance better in the OBE overall?

Consistent with the systematic review by Larwin et al. (2013), we found higher performance on the OBE overall, including on questions testing understanding or application of concepts. We suggest several reasons for this. Firstly, and most simply, it may be possible to find the information required to answer some questions testing understanding or application of concepts using open-book resources, especially the Internet.

Secondly, we suggest that students in CBEs may not be able to answer some higher-order questions because they cannot recall the required information, rather than not being able to understand or apply it. In the OBE, the burden of recall may be reduced, improving performance on higher-order knowledge domains.

Thirdly, OBE performance may have been higher because of reduced anxiety about the exam. We did not measure examinees’ anxiety but we think it is very unlikely that those sitting the OBE felt less anxious, given that they had no experience within the course of performing OBEs and the uncertainty and disruption caused by COVID-19. Furthermore, while examinees may expect themselves to be less anxious in an OBE (Baillie & Toohey, 1997; Broyles et al., 2005), there is evidence that their experienced anxiety is similar (Baillie & Toohey, 1997; Dickson & Miller, 2005).

Finally, students may have prepared differently for the OBE, which could have affected performance (Durning et al., 2016). We did not measure students’ preparation time or approaches. Students were given 6-weeks’ notice of the change in exam format. There had previously been no experience with OBEs within the course. Some evidence suggests students prepare less for OBEs (Agarwal & Roediger, 2011; Boniface, 1985; Moore & Jensen, 2007), though other studies found no difference in preparation time (Betts et al., 2009; Gharib et al., 2012) or study tactics (Broyles et al., 2005; Tamblyn et al., 2007). Importantly, these studies did not use data from summative, end-of-year exams and did not investigate the effects of free access to Internet resources. It will be important in future work to understand how OBEs affect preparation for ‘high-stakes’ assessments like end-of-year exams, and whether knowing they can access the Internet freely affects preparation differently to knowing they can use a course textbook or resources they have made themselves.

## Implications of open-book exams for assessment reliability and validity

Access to external information sources, and especially the Internet, could have significant implications for the reliability and validity of medical school assessments. We consider reliability in general terms as the consistency of measurement. Consistency of measurement over time could be reduced by changes in the resources themselves and variability in examinee’s information-search behaviours across different measurements. The internal consistency of an assessment might be reduced by increased variability in search behaviours across examinees and variability in the availability and quality of information across different domains, such as topics within an assessment. However, this will need to be explored in future studies. The internal consistency of an assessment, particularly as measured by conventional psychometric indices like Cronbach’s alpha (Cronbach, 1951), could actually increase with access to Internet resources, as similarities among search behaviours and resources used by examinees might make their answering patterns more similar.

In terms of validity, allowing examinees to access external resources means that we are no longer only measuring their ability to utilise medical knowledge from their memory, but also their ability to find, evaluate and utilise information acquired through searching. OBEs as assessment tools may therefore be measuring a different set of underlying constructs to a CBE. If we are only interested in measuring an examinee’s ability to use knowledge from their own memory, an OBE could be considered less valid because the influence of search ability on performance could be regarded as irrelevant error. The design of the OBE is likely to influence what proportion of the variability in performance is driven by search ability. The easier it is to search for the answer, the more the assessment is likely to measure search ability.

However, we could also consider OBEs as an opportunity to measure the ability to search for, evaluate and synthesise medical information using the Internet or other resources in a time-pressured environment, as regularly occurs in real-world clinical situations. The ability to efficiently search for information online is an essential skill for modern clinicians, and so is a relevant domain to measure in assessments.

### Future directions for research

Our study raises important questions for future research to better understand how OBEs differ from CBEs and how best to capitalize on their strengths.

Firstly, we did not use remote proctoring. It would be valuable to explore the potential merits of remote proctoring in OBEs.

Secondly, to what extent does giving access to external resources change the validity of assessments, and is the measurement of domains related to information searching relevant to desired outcomes in medical education? Future studies should explore the validity of OBEs within established frameworks such as those of Kane (Kane, 2006) and Messick (Messick, 1984).

Finally, how much will individual differences in examinees’ ability to search for information affect their performance in OBEs? Importantly, does the possibility for differences in search competency risk introducing digital inequality among students, and do medical educators need to design educational activities to develop students’ competencies in information searching?

## Capitalizing on the strengths of open-book exams in medical education

Though we acknowledge there is more to be understood about information-searching in OBEs, this and our previous study (Sam et al., 2020) show that that summative, end-of-year medical school assessments can be conducted as online OBEs without remote proctoring and with free access to Internet resources. Beyond practicality in the COVID-19 era, proponents of OBEs argue they are more authentic to real-world clinical situations encountered by graduates (Durning et al., 2016; Heijne-Penninga et al., 2008; Theophilides & Dionysiou, 1996), promote assessment for learning (Fuller et al., 2020), and move assessment away from assessing factual recall to assessing higher-order domains of knowledge (Lizzio et al., 2002; Schwartzstein & Roberts, 2017; Trigwell & Prosser, 1991). In the wake of the pandemic, medical educators have an opportunity to capitalize on the strengths of OBEs.

We make the following recommendations for educators designing and delivering OBEs:

1. OBEs with free access to resources, including the Internet, are suitable for high-stakes, summative assessments in medical education but question writers should consider how accessing open-book resources, particularly the Internet, will change how examinees answer questions.
2. We recommend delivering OBEs with specialized online platforms that can facilitate delivery of remote, timed assessments and aid in mitigating risks of IT failure, as well as enable safeguards against academic misconduct like randomization of question order and a time-intensive environment.
3. We recommend question-writers consider answering questions in an OBE as an information search task and consider three domains of objective search complexity, following Campbell (Campbell, 1988): 1) what information is required to answer the question, 2) how easy it is to search for it, and 3) what information is available and how easy is it to interpret.
4. To optimize the information required to answer the question, we recommend asking questions that test higher-order domains than factual recall, such as those testing understanding or application of concepts, which can be measured using Bloom’s taxonomy.
5. To optimize how easy it is to search, we recommend question writers reduce the numbers of key terms in the question so that examinees must generate their own search terms, or require examinees to infer key terms for searching based on other information. For example, examinees could be asked a specific question about the pathophysiology of a disease but have to infer what the disease is based on a clinical presentation rather than include the disease name in the question.
6. Regarding interpreting found information, we recommend question-writers try constructing searches based on terms in the question and examine the results in case the search returns unreliable or confusing information.
7. We recommend that educators always bear in mind that an OBE measures search ability as well as ability to recall, understand and apply examinee’s own knowledge. We recommend OBEs are not considered as a general replacement for CBEs. OBEs should be considered as a specific assessment tool that, if well-designed, may measure aspects of the authentic clinical environment that cannot be captured by a CBE. However, if used as a like-for-like replacement for a CBE, an OBE assessment could be less valid due to the impact of an examinee’s search ability.

## Limitations

Our study has some important limitations. The independent variable of whether the exam was closed-book or open-book was not determined intentionally, but effectively assigned by the pandemic. This use of real-world data means that the data are authentic, but there are limitations imposed by factors we had limited ability to control or adjust for.

The OBE and CBE were sat by students at the same stage of training but in different academic cohorts, introducing the potential confounding factor that the cohorts had different ability levels. Given all end-of-year exams were OBEs in 2020 and CBEs in 2019, we cannot exclude this, but some factors may partly mitigate it. Firstly, the selection processes for ICSM was not different in the years these cohorts were selected (2017–2018). Secondly, each cohort was fairly large, with over 300 students, making it less likely that there would be systematic differences in ability levels. Thirdly, the course and teaching content were the same for both years. The 2020 cohort had a shift from face-to-face to online learning from March 16th 2020, but this was after most of their timetabled teaching had taken place.

The exams taken in each year assessed the same topics but had different questions, raising the possibility that a difference in exam difficulty would confound the results. The two exams were standard-set independently using the Ebel procedure and the pass mark was the same in each year, suggesting they had comparable difficulties. The standard-setting for 2020 was performed prior to the conversion to an OBE and prior to conceptualization of this study. 16 of the items were present in both papers.

There is also uncertainty associated with using subjective classification of questions by Bloom’s taxonomy, as well as evidence that students often use different processes than those expected by question-writers (Gierl, 1997). We had acceptable inter-rater reliability in this study but acknowledge the limitation that students may have used different cognitive processes to answer questions.

We have no evidence of academic misconduct in either assessment but also cannot exclude the possibility that differences in exam security could affect our results. We argue this would be unlikely to systematically favour factual recall questions in the OBE and therefore bias our findings.

## Conclusion

We found that free access to open-book resources, including the Internet, in a summative, end-of-year examination for second-year medical students was associated with higher performance overall, in contrast to our previous findings in final-year students (Sam et al., 2020). We found higher performance on questions requiring factual recall than higher-order questions requiring understanding or application of concepts. There was a greater difference in performance on recall versus higher-order questions in the OBE than the CBE, supporting that access to open-book resources confers a particular advantage answering fact-based questions. We theorized this was because the answers to factual recall questions were easier to search for online, because the information required to answer the question is clearer, though this requires further validation. Performance on OBEs is likely to be driven by examinee’s search ability as well as their own medical knowledge, and the difference in what is measured by a CBE and an OBE must be considered when comparing the validity of assessments or choosing which assessment method to use. Understanding objective features of search task complexity will be important when designing OBEs. Understanding individual differences affecting subjective search task complexity will be important to ensure the fairness of OBEs with respect to social inequalities. Some examinees may be systematically disadvantaged if we do not account for factors that affect their search ability, such as digital inequality, which will reduce the validity of an OBE. Our findings support that OBEs can be important assessment tools in medical education, depending on what we are intending to measure.
